# Aggressive Management of a Bilateral Chylothorax Complicating an Orthotopic Heart-Kidney Transplantation

**DOI:** 10.21470/1678-9741-2023-0041

**Published:** 2023-09-19

**Authors:** Breah Lynn Paciotti, Pankaj Garg, Charles A. Ritchie, Kevin Landolfo, Basar Sareyyupoglu

**Affiliations:** 1 Department of Cardiothoracic Surgery, Mayo Clinic Florida, Jacksonville, Florida, United States of America; 2 Department of Radiology, Mayo Clinic Florida, Jacksonville, Florida, United States of America

**Keywords:** Chylothorax, Kidney Transplantation, Kidney, Heart Transplantation

## Abstract

Chylothorax after an orthotopic heart transplant is a rare but potentially detrimental occurrence. This is the first reported case of bilateral chylothorax complicating a heart-kidney transplant patient. No universally accepted protocol exists for the management of chylothorax in general population, let alone the immunocompromised transplant patient. This case presents unique challenges to the management of postoperative chylothorax given heart-kidney transplant’s effect on the patient’s volume status and immunocompromised state. We make the argument for aggressive treatment of chylothorax in an immunocompromised heart-kidney transplant patient to limit complications in a patient population predisposed to infection.

## INTRODUCTION

The first reported case of an orthotopic heart transplant (OHT) complicated by postoperative chylothorax was documented in 1993^[1]^. Since then, few case reports of chylothorax after heart transplant exist; it remains extremely rare^[2,3,4,5,6,7]^. To our knowledge, this is the first reported case of a heart-kidney transplant complicated by postoperative bilateral chylothorax in an adult patient.

A chylothorax is defined as the accumulation of chyle within the pleural space secondary to a disruption to the flow of chyle through the thoracic duct or its tributaries^[8,9]^. While chyle typically appears white or milky, this is observed in less than half of patients with chylous effusions; we should not rely on pleural fluid appearance alone for diagnosis^[8]^.

Trauma, including surgical procedures, accounts for 25% of chylothoraces and remains the second leading cause of chylothorax^[8]^. The incidence of chylothorax among all types of thoracic surgeries is rare; it is reported to occur in 0.2% to 0.5% of the cases^[6]^. The true incidence of chylothorax after an OHT is unknown in the current literature.

It is well-documented that chylothorax management remains a challenge because there are no universally accepted protocols for management^[8,9,10]^. Jacob et al.^[10]^ proposed an algorithm for chylothorax management among lung transplant recipients. Although general treatment recommendations include an initial two-week period of conservative, nonoperative management, there are no validated clinical control trials that support this approach^[8]^. Furthermore, no clear management guidelines exist for chylothorax treatment in the immunosuppressed heart-kidney transplant recipient^[3]^.

## CASE PRESENTATION

A 46-year-old man with a history of non-ischemic cardiomyopathy status post automated implantable cardioverter defibrillator (AICD), congestive heart failure (CHF), and chronic renal failure presented to an outside hospital in cardiogenic shock requiring cardioversion, multiple inotropes, and Impella CP® mechanical circulatory support. After stabilization and diuresis with continuous renal replacement therapy, he was transferred to our institution. We escalated his support to extracorporeal membrane oxygenation (ECMO) and temporary left ventricular assist device (LVAD) Impella 5.5® via right axillary artery cut down. Right ventricular function gradually improved and the ECMO was decannulated. Impella 5.5® support continued for optimal left ventricular unloading. Despite these efforts, he had persistent kidney failure and was listed for combined heart and kidney transplant.

The patient underwent removal of temporary LVAD (Impella 5.5®), AICD, and received an OHT and kidney transplant. His initial postoperative course was unremarkable; he was extubated on postoperative day (POD) 2. Chest tube (CT) output increased over the first postoperative week becoming milky on POD 8. The right pleural CT drained 3000 ml, and the left pleural CT drained 2000 ml in 24 hours. Fluid analysis revealed 453 mg/dL of triglycerides. The patient’s diet was changed to nothing by mouth, and intravenous total parenteral nutrition was started. In the following day, there was increased drainage from the right pleural CT and no change in the left CT output.

**Fig. 1:**
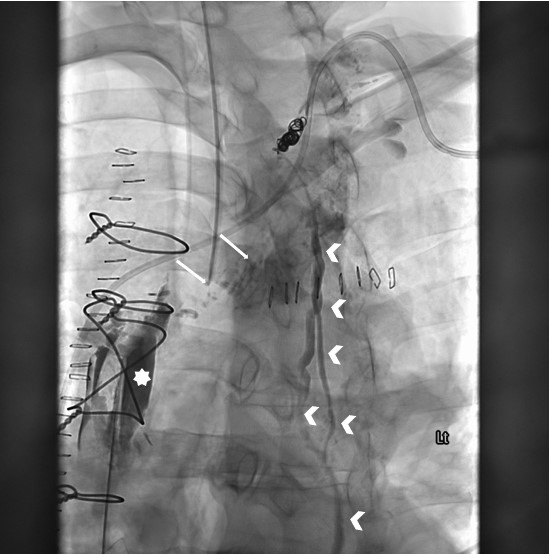
Single anterior-posterior X-ray image from lymphangiogram. Evidence of lymphatic leakage in the anterior mediastinum (*) near the sternotomy from a branch (arrow) of the thoracic duct (arrowhead).

Given the copious CT output, the patient underwent lymphangiogram on POD 10. A large thoracic duct leak was identified in the anterior mediastinum and left pleural space and treated with thoracic duct embolization (Figure 1). In the following day, the serous CT output decreased to 1100 cc from the right CT and 600 cc from the left CT. Patient remained fluid overloaded as the newly transplanted kidney was not functioning optimally immediately after the transplant.

The remainder of his hospital course was uneventful. On POD 30, the patient was discharged to a rehabilitation facility. He has done well without any evidence of rejection or graft dysfunction.

## DISCUSSION

Chylothorax after OHT remains rare, and no guidelines exist for management in this population. This case identifies the importance of timely diagnosis and aggressive treatment of postoperative chylothorax, especially in immunosuppressed transplant patients, to prevent infection and further complications^[2]^.

High CT output should prompt clinical suspicion for chylothorax. Pulle et al.^[9]^ published a retrospective analysis of chylothorax patients in a thoracic surgical unit over eight years. The authors found that excessive pleural drainage (> 1000 ml per day) was an independent predictor of failure of conservative therapy. Our patient had 3000 ml on the right and 2000 ml on the left. Authors obtained pleural triglyceride levels > 110 mg/dL to confirm diagnosis^[1,8]^.

Thoracic duct injuries can present differently based on the location of the injury, with left-sided pleural effusions caused by injury to the thoracic duct above the 5^th^ thoracic vertebra, and right-sided pleural effusions from damage below the 5^th^ thoracic vertebra level^[2]^. The removal of defibrillator leads at the time of OHT can also cause thoracic duct injury leading to pleural effusion. Bowerman et al.^[1]^ proposed that the retraction required during heart transplantation and manipulation of the aorta, pulmonary artery, and other tissues in the thoracic cavity may indirectly injure the lymphatics through stretching.

We believe our patient’s chyle leak was not due to thoracic duct injury as cited in most previous case reports of chylothorax after heart transplant but was instead from two leaking lymphatic channels connected to the thoracic duct. The combined heart and kidney transplant presented challenges with volume overload postoperatively. Persistent systemic venous hypertension secondary to volume overload in the setting of heart transplant and dysfunctional renal allograft led to thoracic duct hypertension likely resulting in small lymphatic channels leaking. After embolization of the thoracic duct, the chyle leak resolved despite the persistent serous drainage from bilateral pleural cavities. CHF patients have eight-fold higher thoracic duct flow, which is exacerbated by renal congestion, and the effects of increased venous pressure on lymphatic drainage in this population are well-known^[11]^. Multiple samples of the output were evaluated for triglyceride levels and were not > 110 mg/dL after embolization. The venous hypertension resolved gradually over the next three weeks as the renal allograft function improved.

Interestingly, the presence of chyle itself in the pleural space is not what predisposes the transplant patient to infection^[8]^. Chyle does not irritate the pleura; it is bacteriostatic. This partially explains why conservative treatment options are often successful, especially when CT output is < 1000 ml per day. The conservative approach to a chylothorax may unfortunately prolong hospitalization and increase morbidity.

Excessive chylous drainage increases risk of dehydration, malnutrition, immune suppression, electrolyte imbalance, metabolic acidosis, lymphocyte, and anti-rejection medication depletion^[1,2,10]^. Chylothorax has also been associated with a higher 30-day risk of sepsis, pneumonia, reintubation, reoperation, and death^[2,10]^. Untreated postoperative chylothorax is associated with mortality rates of up to 82%^[2]^. Surgical thoracic duct ligation has helped decrease mortality from traumatic chylothorax from 50% to nearly 10% since 1948^[8]^. Post-transplant patients are at inherent risk of infection, which may be exacerbated by continued chylous drainage. Berdy et al.^[7]^ identified that chyle leaks can further complicate the management of immunosuppression levels; cyclosporine is secreted in chyle thus decreasing the circulating levels of cyclosporine, which leads to subtherapeutic levels.

## CONCLUSION

We have presented the first case of a chylothorax occurring in an OHT and kidney transplant patient. Increased CT output with or without milky appearance should prompt timely workup for chylothorax. Despite prolonged effusions, we had no evidence of acute rejection or delayed allograft dysfunction. We argue a strong case for aggressive treatment of a postoperative chylothorax with high CT output in an immunocompromised heart-kidney transplant patient to limit further complications and mortality in a patient population predisposed to infection.

## Abbreviations, Acronyms & Symbols

AICD: Automated implantable cardioverter defibrillator
CHF: Congestive heart failure
CT: Chest tube
ECMO: Extracorporeal membrane oxygenation
LVAD: Left ventricular assist device
OHT: Orthotopic heart transplant
POD: Postoperative day
